# Family structure in relation to body mass index and metabolic score in European children and adolescents

**DOI:** 10.1111/ijpo.12963

**Published:** 2022-08-10

**Authors:** Katharina Stahlmann, Lauren Lissner, Leonie H. Bogl, Kirsten Mehlig, Jaakko Kaprio, Joanna C. Klosowska, Luis A. Moreno, Toomas Veidebaum, Antonia Solea, Dénes Molnár, Fabio Lauria, Claudia Börnhorst, Maike Wolters, Antje Hebestreit, Monica Hunsberger, IDEFICS/I.Family consortia

**Affiliations:** ^1^ School of Public Health and Community Medicine, Institute of Medicine, Sahlgrenska Academy University of Gothenburg Göteborg Sweden; ^2^ Institute of Medical Biometry and Epidemiology University Medical Center Hamburg‐Eppendorf Hamburg Germany; ^3^ Department of Epidemiology, Center for Public Health Medical University of Vienna Vienna Austria; ^4^ Institute of Molecular Medicine FIMM University of Helsinki Helsinki Finland; ^5^ Department of Public Health University of Helsinki Helsinki Finland; ^6^ Department of Public Health and Primary Care Ghent University Ghent Belgium; ^7^ GENUD (Growth, Exercise, Nutrition and Development) Research Group, Faculty of Health Sciences, University of Zaragoza Instituto Agroalimenatario de Aragón (IA2) Instituto de Investigación Sanitaria de Aragón Zaragoza Spain; ^8^ Centro de Investigación Biomédica en Red de Fisiopatología de la Obesidad y Nutrición (CIBEROBN) Instituto de Salud Carlos III Madrid Spain; ^9^ Department of Chronic Diseases National Institute for Health Development Tallin Estonia; ^10^ Research and Education Institute of Child Health Strovolos Cyprus; ^11^ Department of Pediatrics, Medical School University of Pécs Pécs Hungary; ^12^ Institute of Food Sciences National Research Council Avellino Italy; ^13^ Department of Biometry and Data Management Leibniz Institute for Prevention Research and Epidemiology – BIPS Bremen Germany; ^14^ Department of Epidemiological Methods and Etiological Research Leibniz Institute for Prevention Research and Epidemiology – BIPS Bremen Germany

**Keywords:** BMI, family structure, metabolic score, only child, overweight, single parent

## Abstract

**Background:**

Living in single parent and blended families or as an only child—compared to living in two‐parent biological families or with siblings, respectively—is associated with a higher body mass index (BMI) in cross‐sectional studies. However, longitudinal research addressing the children's BMI in this context is scarce. Further, little is known about the association between family structure and metabolic health.

**Objectives:**

This study aimed at investigating the association between both aspects of family structure with BMI and a metabolic score (MetS).

**Methods:**

Cross‐sectional data from 7804 children participating in the European multi‐center I.Family study (2013/2014) and longitudinal data from 5621 children who also participated previously in the IDEFICS study (2007–2010) were used. Family structure was assessed by a detailed interview. BMI *z*‐score and the MetS were based on measured anthropometry, blood pressure, high‐density lipoprotein, blood glucose, and triglycerides. Linear regressions were performed to model associations between family structure with BMI and MetS.

**Results:**

Children from single‐parent families had higher BMI *z*‐scores in the cross‐sectional (*β* = 0.09, 95% confidence interval [CI]: 0.001 to 0.18) and longitudinal analyses compared to those from two‐parent families. Cross‐sectionally, the number of siblings was associated with lower BMI *z*‐scores (*β* = −0.07, 95% CI: −0.10 to −0.03) and lower MetS (*β* = −0.14, 95% CI: −0.26 to −0.01). Longitudinally, only children between baseline and follow‐up had higher BMI *z*‐scores at follow‐up (*β* = 0.07, 95% CI: 0.01 to 0.14) compared to stable siblings.

**Conclusion:**

Obesity prevention measures should focus on single‐parent households and families with an only child.

## 1 INTRODUCTION

Childhood overweight and obesity remain a major Public Health concern.^1^ Multiple factors contribute to the development of overweight and obesity, some of which may be related to family structure, which has been changing over the last decades in Western societies. For instance, the share of single‐parent families and of births outside marriage has increased in most EU countries^2^ and ranged from 11% single mothers in Switzerland to 22% in Belgium and from 1% single fathers in Austria and Luxembourg to 15% in Belgium in 2017.^3^ The proportion of one‐child families amounted to 47.5% of all households with children in 2020^2^ with the lowest percentage in Sweden (35.8%) and the highest in Portugal (58.3%) in 2019.^4^

Previous research, including our own in the IDEFICS/I.Family cohort^5^, ^6^, ^7^, ^8^ has examined some aspects of family structure. It has been shown that children living in single‐parent families^6^, ^9^, ^10^, ^11^ or other non‐traditional family structures^6^, ^12^ are at higher risk of having overweight (including obesity) compared to those in two‐parent (biological) families. This may be due to a higher prevalence of obesogenic behaviours in children from these families when compared to two‐parent biological families.^7^, ^13^, ^14^, ^15^, ^16^ Obesogenic behaviours (e.g., low physical activity,^17^ short sleep duration^18^, ^19^ or high screen time^18^, ^19^) and overweight^20^, ^21^ are often associated with the presence of further risk factors for chronic diseases, particularly hypertension, hyperglycemia, dyslipidemia, and abdominal obesity. As these risk factors are inclined to cluster and accumulate, they can be summarized as the metabolic syndrome.^20^ Since family structure is related to overweight and obesity which acts as an important antecedent factor for impaired metabolic health in adulthood,^22^ the question arises whether family structure influences also metabolic health in childhood. Hitherto, research on this outcome is scarce.^8^

Besides parental structure, some studies^5^, ^6^, ^23^, ^24^ reported that being an only child is positively associated with the child's weight. Only children were not only more likely to have overweight or obesity,^5^, ^6^ they also had a higher risk of gaining weight during childhood.^6^, ^25^ Furthermore, other studies observed that only children engage in more obesogenic behaviours.^23^, ^26^, ^27^

To date, most studies have looked at either parental structure or sibling status separately. There are few studies examining both factors simultaneously. For instance, Formisano et al.^6^ observed cross‐sectionally that children with siblings are less likely to have a high body mass index (BMI) than those without siblings and children living with a single‐parent or grandparents are more likely to have a higher BMI. Longitudinally, however, young children without siblings are more likely to develop overweight (or obesity) while parental structure was not associated with overweight.^6^, ^25^ In contrast, Schmeer^9^ reported a higher risk of developing overweight or obesity in children living in stable single‐parent families or with a mother who recently separated compared to those living with stable married parents. Furthermore, compared to children living with a stable single mother, those with mothers who entered a new relationship had a lower BMI.^9^ This evidence shows that both parental structure and sibling status are associated with children's BMI, but these associations differ and it is not clear which factor is more important. Thus, further research is needed to disentangle both aspects of family structure with respect to their association with children's metabolic health to indicate which group should be focused on by prevention measures.

Consequently, this study investigates the association between family structure with BMI and a metabolic score (MetS) by exploring both parental structure and sibling status in European children. To this end, both a cross‐sectional and longitudinal analysis, which followed the children while entering adolescence, were conducted to examine whether findings are consistent in both approaches.

## 2 METHODS

### 2.1 Study sample

This study used data from the multi‐centre IDEFICS/I.Family cohort, which was conducted in eight European countries (Belgium, Cyprus, Estonia, Germany, Hungary, Italy, Spain, and Sweden). All children between 2 and 10 years were eligible (no further exclusion criteria) and, in total, 16 229 children were recruited in school and kindergarten settings for the first wave of the population‐based IDEFICS study (2007–2008) of which 11 041 took also part in a second wave (2009–2010). In addition, 2555 children were newly recruited in the same settings in the second wave. The I.Family study (2013–2014) represented a third wave of the original cohort in which 7118 were reassessed after taking part in either the first and/or the second wave of the IDEFICS study.^28^ To compensate for loss to follow up, 2500 siblings of the IDEFICS children were included in the third wave. Siblings instead of new children were recruited as they are easier to reach and enable a more detailed analysis of family processes, which has been focus of the I.Family study. Detailed information about the cohort, participants' recruitment, and response rates provide Ahrens et al. for the IDEFICS study^28^, ^29^ and for the I.Family study^28^

**FIGURE 1:**
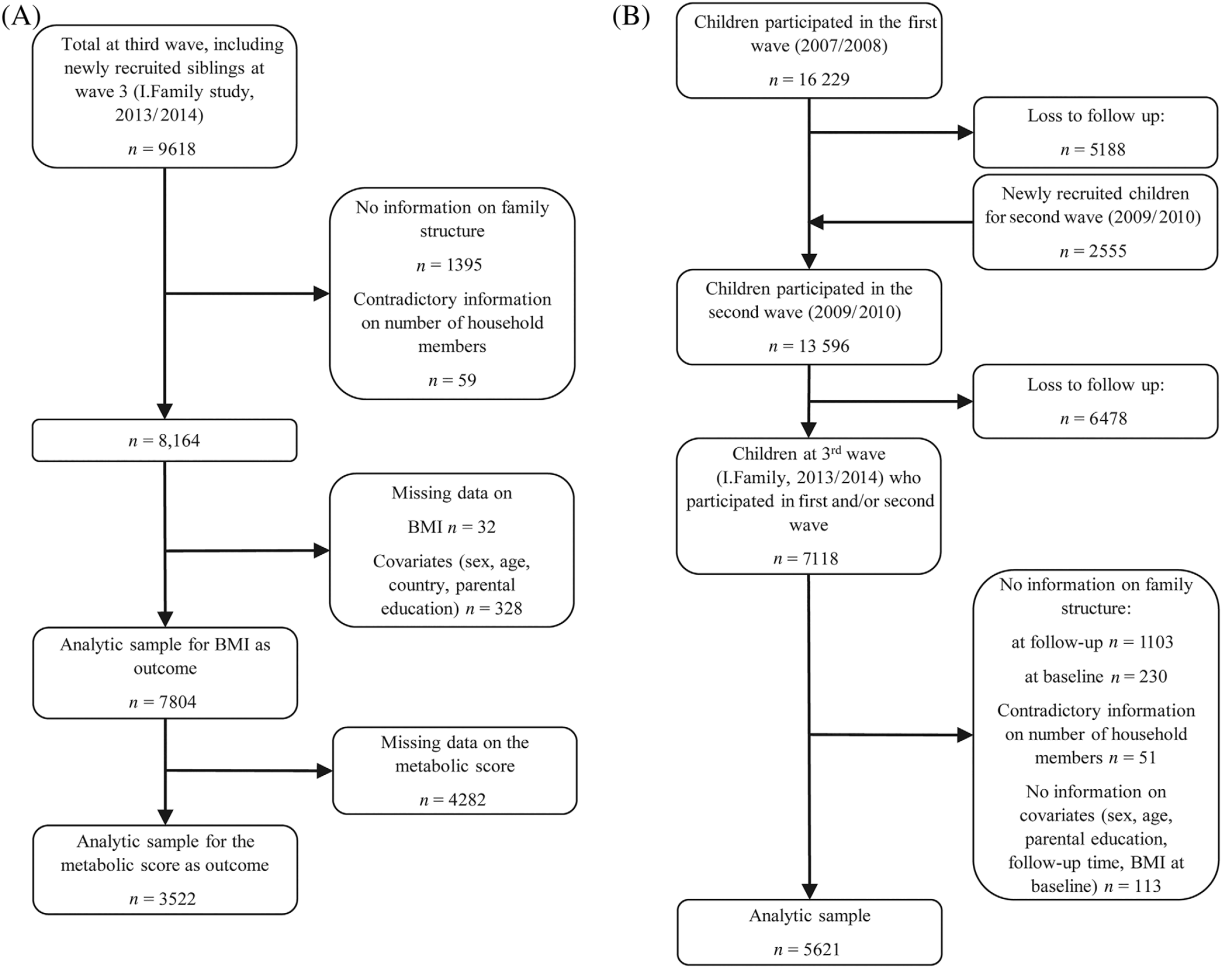
Study flow chart (A) of the cross‐sectional sample (B) of the longitudinal sample

To obtain higher statistical power, the cross‐sectional analysis was based on all data collected during the I.Family study (third wave) including the new siblings who accounted for 25.2% of the final cross‐sectional sample (Figure 1). For the longitudinal analysis, data of the first and second wave were combined to provide baseline data. The second wave was chosen as baseline for those children who had missing data on the variables of interest in the first wave or for those who were newly recruited at the second wave. For all other children, the first wave was set as baseline for our analysis. Thus, the longitudinal analysis examined two time points: first and second wave as baseline and third wave as follow up (Figure 1).

All children aged 2–17 years with valid data on all variables, namely family structure, BMI *z*‐score and covariates, were included in the analysis. Information on family structure was invalid or missing for a few children and led to the exclusion of 1454 (~15.7%) participants from 1031 families and 1384 (~19.9%) participants from 1202 families in the cross‐sectional and in the longitudinal analysis, respectively. Therefore, the cross‐sectional sample comprised 7804 children from 4889 families for the analysis of BMI *z*‐scores as outcome variable. The investigation of the MetS included only 3522 children due to missing data in this variable. The longitudinal analysis examined 5621 children from 4714 families. Before entering the study, parents or legal guardians gave informed consent to the participation of their children and children assented verbally (up to the age of 11 years) or written (12 years and older). Local ethics committees in all eight study centers gave ethical approval of the study.

### 2.2 Family structure

Since the I.Family study (third wave) aimed at exploring family relationships and resemblance more closely, a detailed kinship interview was used to collect information on family structure instead of asking only one question for parental structure and number of siblings each (as in wave 1 and 2, see below). The kinship interview was conducted with the parent or legal guardian via a computer‐assisted telephone call or personal interview.^30^ Along with asking how the interviewed parent is related to the oldest index child (the child who already took part at baseline), the interviewer enquired about the relationship of each household member to the index child. The possible relationship statuses encompassed: biological mother, biological father, biologically unrelated female adult, biologically unrelated male adult, any other adult, biological sibling, half‐sibling, non‐biological sibling.^30^ Using this information, the variable ‘parental structure’ was generated for the cross‐sectional analysis as follows: (a) two‐parent biological family (the child lives with both biological parents and possibly with other household members), (b) single‐parent family (child lives with one parent, either biological, step‐parent or other adult), and (c) blended family (all other family structures, such as living with one biological and one step parent, two step parents or with other adults). The ‘number of siblings’ living in the same household at follow‐up was calculated by summing the number of biological siblings, half‐ and step‐siblings in the household.

For the longitudinal analysis, parental structure at baseline was derived from the question ‘Who does the child live with most of the time?’ with the following response options: (a) with his/her parents, (b) with his/her mother, (c) with his/her mother and her new partner, (d) with his/her father, (e) with his/her father and his new partner, (f) half of the time with his/her mother and the other half with his/her father, (g) with his/her grandparents or other relatives, (h) with foster parents or adoptive parents, (i) in an institution, for example, orphanage, or (j) elsewhere. Based on these responses, the two categories for parental structure at baseline were derived as follows: two‐parent family (biological or non‐biological parents) (a, c, g, h) and single‐parent family (b, d, f). Children living in an institution (*n* = 0) or elsewhere (*n* = 47) were excluded. By comparing parental structure at baseline and at follow‐up (two‐parent biological and blended families collapsed to one category for this purpose due to too few observations in blended families), the variable ‘change in parental structure’ (between baseline and follow‐up) was generated with the following categories: stable single‐parent families, stable two‐parent families (either biological parents or non‐biological), change from single‐parent to two‐parent family, and change from two‐parent to single‐parent family.

The number of siblings at baseline was surveyed with the question ‘How many older and younger siblings does your child live with?’ and dichotomized into none and one or more. Combined with the information on the number of siblings at follow‐up, the variable ‘change in sibling status’ was derived with the categories: stable only children, stable siblings, getting a sibling and becoming an only child in the household (e.g., when an older sibling moves out).

### 2.3 Outcome variables

Age and sex‐specific BMI *z*‐scores calculated based on the International Obesity Task Force criteria (IOTF) by Cole and Lobstein^31^ were the primary outcome variable for the cross‐sectional and longitudinal analysis. These criteria were used as they are internationally accepted and widely used. Thereby, our results are comparable to many studies, including other IDEFICS/I.Family studies which commonly use the IOTF criteria. In comparison, the WHO centile curves are lower before age 6 and higher after age 6. Overall, both curves are similarly shaped.^31^ Therefore, we assume that the trend of our analyses would have been quite similar if we had used the WHO criteria. For the examination, children were asked to wear light clothing and remove their shoes. Weight was measured to the nearest 0.1 kg with a TANITA digital scale (TANITA Europe GmbH, Sindelfingen, Germany) and height to the nearest 0.1 cm by trained field staff using a stadiometer (Seca GmbH & Co. KG., Hamburg, Germany). According to the IOTF, a binary variable distinguishing between having overweight (including obesity) (≥1.31 SD and ≥1.24 SD score equivalent or ≥90.5 and ≥89.3 centile equivalent for boys and girls, respectively, corresponding to a BMI ≥25 at age 18) versus having normal/underweight (<1.31 SD and <1.24 SD score equivalent or <90.5 and <89.3 centile equivalent for boys and girls, respectively, corresponding to a BMI <25 at age 18) was derived.^31^

Criteria for the assessment of the metabolic syndrome among children are not clearly defined^20^ or consistent in the literature.^20^, ^22^ Therefore, this paper utilizes the term ‘metabolic score’ (MetS) instead of metabolic syndrome. The MetS as a composite index based on widely recognized definitions and cut points^20^ of the single metabolic risk factors waist circumference, systolic (SBP) and diastolic (DBP) blood pressure, high‐density lipoprotein (HDL), blood glucose and triglycerides, was examined as the secondary outcome. To calculate the MetS, the single risk factors were standardized to age‐ and sex‐specific (blood pressure additionally for height‐specific) *z*‐scores with a mean of zero and a SD of one according to Ahrens et al.^20^ Reverence values for the age‐ and sex‐specific distribution of the parameters are based on the IDEFICS and I.Family cohort.^21^ *z*‐Scores of all single risk factors were then summed (HDL reversely, multiplied by −1), each score equally weighted, to calculate the composite MetS. The score ranged from −7.4 to 11.9 with higher numbers indicating poorer metabolic health.^20^ Children could opt out from certain examination modules resulting into missing values for the respective risk factors (*n* = 4282 children with missing values on the MetS). The examination procedure has been described in detail elsewhere.^20^, ^21^ A detailed description of statistical modelling and changes in assessment methods can be found in Börnhorst et al.^21^ Owing to a high number of missing values, the MetS was examined in the cross‐sectional analysis only.

### 2.4 Covariates

Sex (male/female), age (in years), and highest parental education level (low, middle and high) according to the International Standard Classification of Education (ISCED)^32^ were selected as covariates in the cross‐sectional analysis based on previous literature.^5^, ^6^, ^8^, ^11^ For the longitudinal analysis, this basic set of covariates was extended by BMI *z*‐score at baseline and follow‐up years to account for baseline differences in the BMI and different follow‐up times between children from wave 1 and wave 2. In one sensitivity analysis, this basic set was further expanded to include baseline behavioural variables (weekly screen time of audiovisual media (in hours), sport club membership (yes/no), and the healthy diet adherence score (continuous)) and psychosocial well‐being^33^ (continuous). See the supplemental material for a detailed description of these variables. Further, parental income level (low, low/medium, medium, medium/high, high) was included in two sensitivity analyses.

### 2.5 Statistical analysis

Descriptive statistics for socio‐demographic variables, family structure, and outcome variables were calculated and expressed as means and 95% confidence interval (CI) for continuous variables and as numbers and percentages for categorical variables. As the data structure is hierarchical with children nested within families and within countries, mixed‐effects linear regression models with family and country as random effects assuming an independent covariance structure were performed to obtain effect estimates and 95% CIs. In the cross‐sectional analysis, parental structure and number of siblings were examined in one linear regression model additionally adjusted for the basic covariates set. We subsequently tested for interactions between parental structure and number of siblings and between exposures and covariates using an interaction term (parental structure * number of siblings; parental structure * covariates; number of siblings * covariates). If the interaction term was statistically significant at *α* = 0.05 level, the sample was stratified and regression estimates and 95% CIs were calculated for these independent subgroups. To explore the robustness of the results, the regression model looking at the MetS as an outcome was further adjusted for the BMI *z*‐score. Owing to many missing values in the variable parental income level, we included this variable as a covariate only in another sensitivity analysis. In a third sensitivity analysis, the sample was restricted to only those children who had taken part in the IDEFICS study (1st and 2nd wave), since siblings were oversampled in the full I.Family study.

The effect of a change in parental structure or in sibling status on BMI *z*‐scores at follow‐up, adjusted for the basic covariates set, was explored in the longitudinal analysis. In a sensitivity analysis, we further adjusted for weekly screen time, healthy diet score, membership in a sports club, and psychosocial wellbeing to examine variables that potentially alter the associations between family structure and health. Second, parental income level was included as covariate in addition to the basic covariates. In the last sensitivity analysis, a mixed logistic regression with country and family as random effects and the binary overweight (including obesity) variable as an outcome was performed to explore whether a change in family structure not only influences the BMI *z*‐scores but also contributes to a change in binary weight status. All statistical calculations were done in Stata 16.1 (Stata Corp, College Station, TX).

## 3 RESULTS

### 3.1 Characteristics of the cross‐sectional study sample

The final cross‐sectional sample had a mean age of 11.0 years (SD ±2.9) and an about equal gender distribution (51.1% boys) (Tables 1 and 2). Most children (82.1%) lived in two‐parent biological families, which was the most common parental structure (78.7% of all families). About 10.0% of all children lived in single‐parent families, which accounted for 11.8% of all families. Blended families, in which 8.0% of all children lived in, made up 9.5% of all families. Parents in 50.1% of two‐parent biological families, in 49.1% of single‐parent families, and in 39.5% of blended families had high educational achievement. While 17.2% of all two‐parent biological and 19.2% of all blended families had a high‐income level, this was true for only 6.4% of all single‐parent families (Table 1). Most children (88.4%) lived with siblings and children with siblings were slightly younger (mean age 10.8, SD ±3.0) than only children (11.6% of all children with a mean age of 11.8, SD ±2.0). Within the full sample, the mean BMI *z*‐score was 0.58 (SD ±1.2) and the mean MetS was 1.1 (SD ±3.1) both of which were higher in only children compared to siblings (Table 2) and showed small differences between parental structures (Table 1).

**TABLE 1:** Descriptive characteristics of the cross‐sectional sample, stratified by parental structure

	Two‐parent biological family	Single‐parent family	Blended family	Full sample
On family level				
*n* (%)	3848 (78.71)	578 (11.82)	463 (9.47)	4889 (100)
Maternal age, *n*	3281	445	365	4091
Mean ± SD (95% CI)	41.11 ± 5.21 (40.93 to 41.29)	41.31 ± 5.96 (40.76 to 41.87)	39.13 ± 6.18 (38.50 to 39.77)	40.96 ± 5.42 (40.79 to 41.12)
Paternal age, *n*	3288	367	331	3986
Mean ± SD (95% CI)	44.11 ± 5.86 (43.91 to 44.31)	43.67 ± 6.55 (43.00 to 44.34)	42.05 ± 6.77 (41.32 to 42.78)	43.90 ± 6.03 (43.71 to 44.08)
Parental education, *n* (%)				
Low	215 (5.59)	36 (6.23)	35 (7.56)	286 (5.85)
Medium	1707 (44.36)	258 (44.64)	245 (52.92)	2210 (45.20)
High	1926 (50.05)	284 (49.13)	183 (39.52)	2393 (48.95)
Parental income, *n* (%)				
Low income	612 (15.90)	185 (32.01)	68 (14.69)	865 (17.69)
Low/medium income	275 (7.15)	68 (11.76)	38 (8.21)	381 (7.79)
Medium income	1129 (29.34)	158 (27.34)	146 (31.53)	1433 (29.31)
Medium/high income	428 (11.12)	25 (4.33)	45 (9.72)	498 (10.19)
High income	743 (17.18)	37 (6.40)	89 (19.22)	869 (17.77)
Missing	661 (17.18)	105 (18.17)	77 (16.63)	843 (17.24)
Country, *n* (%)				
Italy	862 (22.40)	42 (7.27)	32 (6.91)	936 (19.15)
Estonia	313 (8.13)	75 (12.98)	114 (24.62)	502 (10.27)
Cyprus	985 (25.60)	98 (16.96)	40 (8.64)	1123 (22.97)
Belgium	61 (1.59)	12 (2.08)	12 (2.59)	85 (1.74)
Sweden	408 (10.60)	71 (12.28)	40 (8.64)	519 (10.62)
Germany	482 (12.53)	122 (21.11)	86 (18.57)	690 (14.11)
Hungary	518 (13.46)	121 (20.93)	121 (26.13)	760 (15.55)
Spain	219 (5.69)	37 (6.40)	18 (3.89)	274 (5.60)
On individual level				
*n* (%)	6403 (82.05)	778 (9.97)	623 (7.98)	7804 (100)
Age, mean ± SD (95% CI)	10.84 ± 2.93 (10.77 to 10.92)	11.50 ± 2.58 (11.32 to 11.68)	11.30 ± 2.80 (11.08 to 11.52)	10.95 ± 2.89 (10.88 to 11.01)
Sex, *n* (%)				
Male	3275 (51.15)	396 (50.90)	316 (50.72)	3987 (51.09)
Female	3128 (48.85)	382 (49.10)	307 (49.28)	3817 (48.91)
Number of siblings, *n* (%)				
0	565 (8.82)	225 (28.92)	116 (18.62)	906 (11.61)
1	3506 (54.76)	388 (49.87)	233 (37.40)	4127 (52.88)
2	1767 (27.60)	138 (17.74)	185 (29.70)	2090 (26.78)
3 or more	565 (8.82)	27 (3.47)	89 (14.29)	681 (8.73)
Outcomes				
BMI *z*‐score, mean ± SD (95% CI)	0.59 ± 1.15 (0.56 to 0.62)	0.56 ± 1.16 (0.48 to 0.64)	0.49 ± 1.13 (0.40 to 0.58)	0.58 ± 1.15 (0.55 to 0.60)
MetS, *n*	2873	353	296	3522
Mean ± SD (95% CI)	1.03 ± 3.13 (0.91 to 1.14)	1.03 ± 3.15 (0.70 to 1.36)	1.35 ± 3.25 (0.98 to 1.72)	1.06 ± 3.14 (0.95 to 1.16)

Abbreviations: BMI, body mass index; CI, confidence interval; MetS, metabolic score; *n*, number of observations.

**TABLE 2:** Descriptive characteristics of the cross‐sectional sample on child level, stratified by sibling status

	Only child	Sibling	Full sample
*n* (%)	906 (11.61)	6898 (88.39)	7804 (100)
Age, mean ± SD (95% CI)	11.75 ± 2.01 (11.62 to 11.88)	10.84 ± 2.97 (10.77 to 10.91)	10.95 ± 2.89 (10.88 to 11.01)
Sex, *n* (%)			
Male	462 (51.99)	3525 (51.10)	3987 (51.09)
Female	444 (49.01)	3373 (48.90)	3817 (48.91)
Parental education, *n* (%)			
Low	43 (4.75)	400 (5.80)	443 (5.68)
Medium	447 (49.34)	3029 (43.91)	3476 (44.54)
High	416 (45.92)	3469 (50.29)	3885 (49.78)
Country, *n* (%)			
Italy	138 (15.23)	1289 (18.69)	1427 (18.29)
Estonia	125 (13.80)	554 (8.03)	679 (8.70)
Cyprus	149 (16.45)	2065 (29.94)	2214 (28.37)
Belgium	4 (0.44)	145 (2.10)	149 (1.91)
Sweden	55 (6.07)	721 (10.45)	776 (9.94)
Germany	180 (19.87)	944 (13.69)	1124 (14.40)
Hungary	188 (20.75)	871 (12.63)	1059 (13.57)
Spain	67 (7.40)	309 (4.48)	376 (4.82)
Outcomes			
BMI *z*‐score, mean ± SD (95% CI)	0.74 ± 1.12 (0.67 to 0.81)	0.56 ± 1.15 (0.53 to 0.58)	0.58 ± 1.15 (0.55 to 0.60)
MetS, *n*	493	3029	3522
Mean ± SD (95% CI)	1.66 ± 3.11 (1.39 to 1.94)	0.96 ± 3.14 (0.85 to 1.07)	1.06 ± 3.14 (0.95 to 1.16)

Abbreviations: BMI, body mass index; CI, confidence interval; MetS, metabolic score; *n*, number of observations.

### 3.2 Cross‐sectional association between family structure metabolic health

Compared to those living in two‐parent biological families, children from single‐parent families were more likely to have a higher BMI *z*‐score (*β* = 0.09, 95% CI: 0.001 to 0.18) (Table 3). The interaction term between parental structure and sex of the child (*p* = 0.008) and subgroup analyses indicated sex differences in this association. Girls from blended families were more likely to have higher BMI *z*‐scores (*β* = 0.19, 95% CI: 0.05 to 0.32) than those in two‐parent biological families, whereas no association was seen in boys. A greater number of siblings was associated with lower BMI *z*‐scores (*β* = −0.07 per additional sibling, 95% CI: −0.10 to −0.03) and this association did not differ between sexes (Table 3). There was no significant interaction between the parental structure and the number of siblings or between exposures and other covariates (age, education) apart from sex of the child. Adjusting for parental income attenuated the results for parental structure so that the association of living in single‐parent families with BMI *z*‐scores became non‐significant (Table S1). The restriction to only IDEFICS children did not alter the main results (Table S1).

**TABLE 3:** Cross‐sectional association between family structure and children's body mass index *z*‐score in the full sample and sex‐stratified subgroups

	Full sample *n* = 7804		Boys *n* = 3987		Girls *n* = 3817	
	*β* (95% CI)	*p*‐Value	*β* (95% CI)	*p*‐Value	*β* (95% CI)	*p*‐Value
Parental structure^a^						
Two‐parent bio. family	Ref.		Ref.		Ref.	
Single‐parent family	0.09 (0.001 to 0.18)	0.048	0.06 (−0.06 to 0.18)	0.53	0.11 (−0.02 to 0.23)	0.08
Blended family	0.07 (−0.04 to 0.17)	0.20	−0.07 (−0.21 to 0.06)	0.29	0.19 (0.05 to 0.32)	0.006
Number of siblings^a^	−0.07 (−0.10 to −0.03)	<0.001	−0.07 (−0.11 to −0.02)	<0.001	−0.06 (−0.11 to −0.02)	0.005

Abbreviations: *β*, effect estimate; bio., biological; CI, confidence interval; Ref., reference category.

^a^ Mixed‐effects linear regression model with parental structure, number of siblings, sex (excluded in sex‐stratified subgroups), age, parental education as independent variables, while country and family are random effects. The results are shown only for parental structure and number of siblings.

Overall, results on the MetS were mostly consistent with the results for BMI *z*‐scores (Tables S2 and S3). In contrast to results for BMI *z*‐score, however, there was an interaction between parental structure and the number of siblings (*p* = 0.001). The number of siblings was negatively associated with the MetS in children from single‐parent and blended families but not in those from two‐parent biological families (Table S2). Furthermore, living in single‐parent families was associated with a higher MetS compared to two‐parent biological families among only children but not siblings (Table S3). The association between the number of siblings and the MetS became non‐significant and near a null‐effect in the regression model additionally adjusted for BMI *z*‐scores (Table S4).

### 3.3 Characteristics of the longitudinal sample

Table 4 and Table S5 show the baseline characteristics of the longitudinal study sample (mean age 6.0, SD ±1.9 and 51.0% male). Most children did not experience a change in sibling status and lived as stable siblings (78.6%) or stable only child (11.5%) between baseline and follow‐up. A change from living as an only child to having one or more siblings or from being a sibling to being an only child was experienced by 6.0% and 3.9%, respectively. While 5.1% of all children lived in stable single‐parent families and 5.9% changed from living in a two‐parent family to a single‐parent family, most children lived in stable two‐parent families (76.5%) or experienced a change from single‐parent to two‐parent family (12.6%). Half of all children had parents with high education, with higher proportions in two‐parent families at baseline. The BMI *z*‐score was higher in children living in single parent families (mean 0.8 SD ±1.2) and in only children (0.7 SD ±1.1) compared to those living in two‐parent families (0.5 SD ±1.1) or with siblings (0.6 SD ±1.1) at baseline, respectively.

**TABLE 4:** Descriptive characteristics of the longitudinal sample, stratified by baseline family structure

Baseline characteristics	Sibling status at baseline		Parental structure at baseline		Full sample
	Only children	Siblings	Single‐parent family	Two‐parent family	
*n* (%)	983 (17.49)	4638 (82.51)	995 (17.70)	4626 (82.30)	5621 (100)
Age, mean ± SD (95% CI)	5.70 ± 1.87 (5.58 to 5.83)	6.11 ± 1.85 (6.06 to 6.16)	6.21 ± 1.84 (6.10 to 6.32)	6.00 ± 1.88 (5.95 to 6.06)	6.04 ± 1.87 (5.99 to 6.09)
Sex, *n* (%)					
Male	518 (52.70)	2347 (50.60)	529 (53.17)	2336 (50.50)	2865 (50.97)
Female	465 (47.30)	2291 (49.40)	466 (46.83)	2290 (49.50)	2756 (49.03)
Parental education, *n* (%)					
Low	65 (6.61)	279 (6.02)	109 (10.95)	235 (5.08)	344 (6.12)
Medium	472 (48.02)	2033 (43.83)	524 (52.66)	1981 (42.82)	2505 (44.57)
High	446 (45.37)	2326 (50.15)	362 (36.38)	2410 (52.10)	2772 (49.32)
Parental income, *n* (%)					
Low income	241 (24.52)	853 (18.39)	393 (39.50)	701 (15.15)	1094 (18.46)
Low/medium income	173 (17.60)	760 (16.39)	215 (21.61)	718 (15.52)	933 (16.60)
Medium income	217 (22.08)	1109 (23.91)	153 (15.38)	1173 (25.36)	1326 (23.59)
Medium/high income	130 (13.22)	685 (14.77)	83 (8.34)	732 (15.82)	815 (14.50)
High income	176 (17.90)	952 (20.53)	83 (8.34)	1045 (22.59)	1128 (20.07)
Missing	46 (4.68)	279 (6.02)	68 (6.83)	257 (5.56)	325 (5.78)
Country, *n* (%)					
Italy	194 (19.74)	909 (19.60)	311 (31.26)	792 (17.12)	1103 (19.62)
Estonia	156 (15.87)	372 (8.02)	83 (8.34)	445 (9.62)	528 (9.39)
Cyprus	120 (12.21)	1087 (23.44)	297 (29.85)	910 (19.67)	1207 (21.47)
Belgium	7 (0.71)	110 (2.37)	4 (0.40)	113 (2.44)	117 (2.08)
Sweden	71 (7.22)	634 (13.67)	45 (4.52)	660 (14.27)	705 (12.54)
Germany	182 (18.51)	626 (13.50)	110 (11.06)	698 (15.09)	808 (14.37)
Hungary	182 (18.51)	672 (14.49)	123 (12.36)	738 (15.95)	861 (15.32)
Spain	64 (6.51)	228 (4.92)	22 (2.21)	270 (5.84)	292 (5.19)
BMI *z*‐score at baseline, mean ± SD (95% CI)	0.41 ± 1.22 (0.34 to 0.49)	0.38 ± 1.18 (0.34 to 0.41)	0.58 ± 1.24 (0.50 to 0.65)	0.34 ± 1.17 (0.31 to 0.38)	0.38 ± 1.19 (0.35 to 0.41)
Outcome at follow up					
BMI *z*‐score, mean ± SD (95% CI)	0.70 ± 1.12 (0.63 to 0.77)	0.57 ± 1.14 (0.53 to 0.60)	0.83 ± 1.16 (0.76 to 0.90)	0.54 ± 1.12 (0.51 to 0.57)	0.59 ± 1.14 (0.56 to 0.62)
Sibling status at follow‐up					
Only child	644 (11.46)	219 (3.90)	—	—	863 (15.35)
Siblings	339 (6.03)	4419 (78.62)	—	—	4758 (84.65)
Parental structure at follow‐up					
Single‐parent family	—	—	289 (5.14)	329 (5.85)	995 (17.70)
Two‐parent family	—	—	706 (12.56)	4297 (76.45)	4626 (82.30)

Abbreviations: BMI, body mass index; CI, confidence interval; *n*, number of observations.

### 3.4 Longitudinal associations between changes in family structure and BMI

Results of the longitudinal analysis matched the results of the cross‐sectional analysis (Table 5). Compared to children in stable two‐parent families, those living in stable single‐parent families had higher BMI *z*‐scores at follow‐up (*β* = 0.10, 95% CI: 0.01 to 0.19). In reference to children living with siblings at baseline and follow‐up, only children at both time points and children who changed from being a sibling to being an only child had higher BMI *z*‐scores at follow up (*β* = 0.07, 95% CI: 0.01 to 0.14 and *β* = 0.13, 95% CI: 0.03 to 0.23, respectively).

**TABLE 5:** Longitudinal association of a change in parental structure and in sibling status with children's body mass index (BMI) *z*‐scores at follow‐up

	Full sample basic covariates set^a^ *n* = 5621		Further adjusted for behaviour factors/wellbeing^b^ *n* = 4591		Further adjusted for income^c^ *n* = 5296	
	*β* (95% CI)	*p*‐Value	*β* (95% CI)	*p*‐Value	*β* (95% CI)	*p*‐Value
Change in parental structure						
Stable two‐parent family	Ref.		Ref.		Ref.	
Stable single‐parent family	0.10 (0.01 to 0.19)	0.032	0.10 (−0.01 to 0.20)	0.06	0.05 (−0.05 to 0.15)	0.30
Change from two to single parent family	0.07 (−0.02 to 0.15)	0.13	0.08 (−0.01 to 0.17)	0.10	0.05 (−0.03 to 0.14)	0.24
Change from single to two‐parent family	0.02 (−0.04 to 0.08)	0.48	0.02 (−0.05 to 0.09)	0.64	0.003 (−0.06 to 0.07)	0.92
Change in sibling status						
Stable sibling	Ref.		Ref.		Ref.	
Stable only child	0.07 (0.01 to 0.14)	0.021	0.08 (0.01 to 0.15)	0.017	0.08 (0.01 to 0.15)	0.017
Getting a sibling	0.07 (−0.01 to 0.16)	0.08	0.09 (0.004 to 0.18)	0.041	0.08 (−0.004 to 0.17)	0.060
Becoming an only child	0.13 (0.03 to 0.23)	0.011	0.13 (0.02 to 0.25)	0.021	0.11 (0.01 to 0.22)	0.036

Abbreviations: *β*, effect estimate; CI, confidence interval; Ref., reference category.

^a^ Mixed‐effects linear regression model with change in parental structure, change in sibling status, sex, age at baseline, parental education, BMI *z*‐score at baseline and follow‐up time as independent variables, while country and family are random effects. The results are shown only for parental structure and number of siblings.

^b^ Mixed‐effects linear regression model with change in parental structure, change in sibling status, sex, age at baseline, parental education, BMI *z*‐score at baseline, follow‐up time, weekly screen time, membership in a sports club, fat score, fruit/vegetable score, sweet score, healthy diet score, wellbeing (behavioural & wellbeing variables at baseline) as independent variables, while country and family are random effects. The results are shown only for parental structure and number of siblings.

^c^ Mixed‐effects linear regression model with change in parental structure, change in sibling status, sex, age at baseline, parental education, parental income, BMI *z*‐score at baseline and follow‐up time as independent variables, while country and family are random effects. The results are shown only for parental structure and number of siblings.

Additional adjustment for behavioural variables and wellbeing at baseline did not alter the main effect estimates substantially (Table 5). In line with results of the cross‐sectional sensitivity analysis, the effect estimates for living in single‐parent families was lower and became non‐significant when adjusting for parental income (Table 5). Moreover, the mixed logistic regression with the binary overweight/obesity outcome variable confirmed the results of the linear regression on parental structure. In contrast to the main analysis, no association between a change in sibling status and BMI status and no significant interaction could be detected in the logistic model (Table S6).

## 4 DISCUSSION

The cross‐sectional analysis revealed that children living in single‐parent families and girls from blended families were more likely to have higher BMI *z*‐scores than those living in two‐parent biological families. Furthermore, BMI *z*‐scores were lower in children with siblings. This was also true for the MetS, which was negatively related to the number of siblings in single‐parent and blended families and positively associated with living in single‐parent families in only children. However, it was mostly explained by the association with BMI and the respective sample sizes were small. Therefore, the results of the MetS analysis should be interpreted with caution due to the low power and the high margin of error. Longitudinal results confirmed the cross‐sectional results. These associations did not change even when controlling for behavioural factors and wellbeing at baseline but adjusting for parental income attenuated the association with parental structure.

Overall, the findings of this study are in line with previous cross‐sectional and longitudinal research that observed a higher risk of overweight and a higher BMI in children from single‐parent families^6^, ^9^, ^10^, ^11^ and other non‐traditional families.^6^, ^12^ Along with earlier IDEFICS studies^5^, ^6^ and a recent systematic review,^34^ we showed that living with siblings was associated with a lower BMI *z*‐score. Our study extends the current state of research by indicating that family structure is associated with the MetS which is, however, explained by the association with the BMI. Compared to a previous IDEFICS study^8^ who reported a generally higher risk for worse metabolic health in children from non‐traditional families, the current study observed a higher MetS merely in only children from single‐parent families compared to those from two‐parent biological families. This may be because Iguacel et al.^8^ performed a longitudinal analysis using only data from the first and second waves. Thus, children in our study were older compared to the sample of Iguacel et al.^8^ A probable explanation for the association between family structure and BMI is the higher prevalence of obesogenic behaviours in children living in non‐traditional families or as an only child.^34^ Compared to children from two‐parent (biological) families or children living with siblings, they are more likely to have higher screen times,^7^ including more television watching,^26^ to consume less fruit and vegetables,^13^, ^14^, ^26^ but more fast food^24^ and sugar sweetened beverages^7^, ^13^, ^14^, ^26^ and less likely to be physical active.^15^, ^16^, ^27^ Nonetheless, adjustment for behavioural factors and wellbeing at baseline did not alter the longitudinal association between family structure and BMI. It is possible that these factors are not so decisive at baseline but change over time. To a certain extent, obesogenic behaviours are influenced by underlying parental processes, such as parental food habits^35^ or family rules.^7^ With respect to parental structure, children in non‐traditional families may face several challenges that can act as risk factors for worse health and wellbeing such as lower social support and parental financial and time‐related stress (in case of single‐parent families).^36^ They also tend to experience a higher level of family conflict^37^ as well as a psychological burden due to the parents' separation.^38^ In turn, children in families with higher conflict may engage in more obesogenic behaviours and, thus, may be more likely to have a higher BMI.^37^, ^39^ Furthermore, other processes are also relevant in shaping this relationship. For instance, some of the association between family structure and BMI/obesogenic behaviours is mediated by income,^11^, ^14^, ^16^ as especially single‐parent families are burdened by financial constraints.^40^ Thus, they lack the means to enrol their children in a sports club,^16^ among others. Our sensitivity analyses supported the fact that parental income is an explaining factor in the association between parental structure and BMI.

In regard of sibling status, one reason for the lower prevalence of obesogenic behaviours in siblings compared to only children may be that they have each other to play with. While older children encourage physically active activities, younger children encourage free play in their siblings.^41^ Nevertheless, a larger birth spacing between siblings is associated with a higher risk of overweight in the younger sibling, who are, generally, more likely to have overweight or obesity compared to older ones.^23^, ^24^, ^32^ This may be because parents are less vigilant in their younger children^34^ and that older children may also act as role models in terms of engaging in higher screen times.^34^, ^41^ In addition, Bogl et al.^42^ observed that near‐aged siblings' behaviour is more similar than the behaviour of siblings with a larger age gap. Furthermore, fast food consumption and screen time behaviour were more similar between younger siblings, while these behaviours were more similar with the behaviour of peers than siblings in older children.^42^

In summary, our findings show that parental structure and sibling status are independently associated with children's BMI. Hence, public health actions should focus particularly on supporting non‐traditional families and only children in maintaining a healthy weight. In contrast to controlling for parental education, adjustment for parental income attenuated the effect estimates for parental structure. Therefore, financial support should be provided to non‐traditional families as they may suffer from more time and financial burden. In addition, only children and children living in non‐traditional families may need more access to playtime and should be encouraged to reduce screen times. For instance, it has been recommended that parents should impose rules on screen time and the consumption of unhealthy foods as the presence of such rules are associated with less obesogenic behaviours.^7^, ^43^ Parents can also promote a healthy lifestyle by acting as role models for their children.^35^, ^43^ In addition to the home environment, kindergartens and schools play an essential part in the child's health. For instance, improvements in the nutritional balance of school meals in the US have led to a reduction of overweight and obesity in school‐aged children.^44^ Therefore, it is necessary to make school meals affordable for all children by, for instance, introducing free school meals as it is the case in the Nordic countries.^45^ Moreover, the enrollment in sports clubs would benefit particularly only children by promoting their physical activity and overall fitness.^46^ In addition, doing sport activities and participation in other types of extracurricular activities, such as community or arts programs, elevates mental wellbeing through higher levels of social support^47^ and reduces screen time.^48^ At last, intervention programs should not only target the child but involve the whole family since multiple processes within the family influence the child's behaviour and weight.^49^ Besides addressing unhealthy lifestyles within the family, their cohesion and the child's resilience against family conflicts should be improved.

### 4.1 Limitations and strengths

Our study has some limitations. First, information on family structure was invalid or missing for a few children and possibly misclassified for other children since the question wording for the assessment of family structure changed between baseline and follow‐up. We do not assume that answers regarding family structure in the third wave were biased in the telephone kinship interview compared to the personal interview since this is not such a sensitive topic. Previous research showed that responses on socio‐demographic characteristics do not differ between both modes.^50^ Data on the MetS were available for a smaller sample in the cross‐sectional analysis since the examination was voluntary and some children chose to opt out. Overall, a response rate of 51% was obtained^29^ in the first wave by employing extended recruitment efforts with multiple reminders to the participants.^51^ Late responders were more likely to have a low socioeconomic status.^51^ Participants who dropped out in the course of the study were slightly more likely to have overweight or obesity and to have parents with low/medium education than those staying in the study.^52^ Nonetheless, the distribution of the BMI and estimates of associations between socio‐economic characteristics, obesogenic behaviours and BMI differed only slightly between subgroups with different attrition pattern.^52^ Additionally, two‐parent biological families were oversampled and, therefore, non‐traditional families slightly underrepresented in the I.Family study.^30^ Hence, effect estimates might be slightly underestimated due to selection bias. Finally, the presence of residual confounding owing to imprecise measurement or unmeasured variables cannot be excluded. Obesogenic behaviours may be influenced by social desirability or recall bias which could lead to an underestimation of their effects. Nevertheless, our main outcome variables—BMI and MetS—are likely to be correctly measured since physical examinations were performed based on highly standardized and quality‐assured protocols and by trained field‐staff. Furthermore, the MetS considered the age‐ and sex‐specific distribution of its parameters, whose estimates are valid for a heterogeneous, large, and unselected population of healthy children.^20^ Another strength is the large international sample representing multiple European countries and the detailed kinship interview at follow‐up. Our study provides insights into objectively measured metabolic health in children which has been rarely examined in the current literature. Moreover, few studies have looked at the two main exposures, parental structure and sibling status, simultaneously. Further, several sensitivity analyses were conducted that explored the associations more deeply.

### 4.2 Conclusion

This study showed that both parental structure and sibling status are associated with children's BMI and metabolic health. Especially children living in single‐parent families and only children have an elevated risk of a high BMI independent of parental education. However, parental income mainly explained the relationship with parental structure. Consequently, overweight prevention measures should be target children living in non‐traditional families and those without siblings. It might be desirable to design intervention trials focused on these high‐risk families.

## AUTHOR CONTRIBUTIONS

Lauren Lissner, Jaakko Kaprio, Monica Hunsberger, Leonie H. Bogl, Kirsten Mehlig, and Katharina Stahlmann conceptualized the study, analysed the data and drafted the manuscript. Lauren Lissner, Leonie H. Bogl, Kirsten Mehlig, Jaakko Kaprio, Joanna C. Klosowska, Luis A. Moreno, Toomas Veidebaum, Antonia Solea, Dénes Molnár, Fabio Lauria, Claudia Börnhorst, Maike Wolters, Antje Hebestreit, and Monica Hunsberger contributed to the data collection. All authors contributed to the manuscript and approved the manuscript.

## CONFLICT OF INTEREST

The authors declare that they have no competing interests.

## Supporting information


**Table S1.** Supporting information.
