# Supplementary material for: Family structure in relation to body mass index and metabolic score in European children and adolescents
Source: Pediatr Obes. 2022 Aug 10;17(12):e12963. doi: 10.1111/ijpo.12963 (PMC9786348; doi:10.1111/ijpo.12963)
Supplement: Supplementary file 1 — Table S1. Supporting information. [file IJPO-17-e12963-s001.pdf]

Family structure in relation to BMI and metabolic score in European children and adolescents

Katharina Stahlmann[1,2], Lauren Lissner[1], Leonie H. Bogl[3,4], Kirsten Mehlig[1], Jaakko Kaprio[4,5], Joanna C. Klosowska[6], Luis A. Moreno[7,8], Toomas Veidebaum[9], Antonia Solea[10], Dénes Molnár[11], Fabio Lauria[12], Claudia Börnhorst[13], Maike Wolters[13], Antje Hebestreit[13], and Monica Hunsberger[1] on behalf of the IDEFICS/I.Family consortia

[1]School of Public Health and Community Medicine, Institute of Medicine, Sahlgrenska Academy, University of Gothenburg, Sweden

[2]Institute of Medical Biometry and Epidemiology, University Medical Center Hamburg-Eppendorf, Hamburg, Hamburg, Germany

[3]Department of Epidemiology, Center for Public Health, Medical University of Vienna, Vienna, Austria

[4]Institute of Molecular Medicine FIMM, University of Helsinki, Helsinki, Finland

[5]Department of Public Health, University of Helsinki, Helsinki, Finland

[6]Department of Public Health and Primary Care, Ghent University, Ghent, Belgium

[7]GENUD (Growth, Exercise, Nutrition and Development) Research Group, Faculty of Health Sciences, University of Zaragoza Instituto Agroalimenatario de Aragón (IA2), Instituto de Investigación Sanitaria de Aragón, Zaragoza, Spain

[8]Centro de Investigación Biomédica en Red de Fisiopatología de la Obesidad y Nutrición (CIBEROBN), Instituto de Salud Carlos III, Madrid, Spain

[9]Department of Chronic Diseases, National Institute for Health Development, Tallin, Estonia

[10]Research and Education Institute of Child Health, Strovolos, Cyprus

[12]Department of Pediatrics, Medical School, University of Pécs, Pécs, Hungary

[12]Institute of Food Sciences, National Research Council, Avellino, Italy

[13]Leibniz Institute for Prevention Research and Epidemiology – BIPS, Bremen, Germany

Corresponding author:

Katharina Stahlmann

University of Gothenburg

School of Public Health and Community Medicine

Box 463

SE 405 30 Göteborg

Katharina.Stahlmann@web.de

Supplemental material

Description of additional covariates in the longitudinal analysis

In a sensitivity analysis, the covariates for the longitudinal analysis were extended by the following variables: weekly screen time (continuously in hours), membership in a sports club (yes/no), the healthy diet adherence score (HDAS) and the KINDL well-being score. Weekly screen time was calculated based on information on the use of audiovisual media (television, video/DVD, computer, and gaming console) on weekdays and weekends (in hours). Information on diet were collected via the Children's Eating Habits Questionnaire which had been filled out by the parents and included a validated 43-item food frequency questionnaire (FFQ). The parents were asked to report their child's consumption of selected foods on a typical day, except for those consumed in school or childcare settings. Children were excluded when they had missing data for more than 50% of the FFQ items. Validity and reproducibility of the FFQ have been described in detail elsewhere[1]. The HDAS is intended to reflect the adherence to healthy dietary guidelines based on recommendations of Waijers et al.[2] and consists of five components: whole meal, fruits/vegetables, fat, sugar and fish. By summing the values of each component, with individual scores ranges from 0 to 10, the HDAS is calculated with a range from 0 to 50. A higher score value indicate a stronger adherence to a healthy diet. More detailed information on the HDAS and its components can be found in Arvidsson et al.[3]. To capture the child's psychosocial well-being, the IDEFICS/I.Family study employed a version of the validated Kinder "Lebensqualität Fragebogen" (KINDL)[4,5] in which parents responded on behalf of children and adolescents aged 7 to 17. The questionnaire included the four dimensions emotional well-being, self-esteem, parent relations and social contacts with four items for each dimension and answer categories ranging from 0 (not at all) to 3 (often or always). After summing the responses within each dimension (reversing items when necessary), a composite score was calculated with values between 0 (low psychosocial well-being) and 48 (high psychosocial well-being).

References

1. Lanfer A, Hebestreit A, Ahrens W, et al. Reproducibility of food consumption frequencies derived from the Children's Eating Habits Questionnaire used in the IDEFICS study. *Int J Obes (Lond).* 2011;35 Suppl 1:S61-68.
2. Waijers PM, Feskens EJ, Ocké MC. A critical review of predefined diet quality scores. *Br J Nutr.* 2007;97(2):219-231.
3. Arvidsson L, Eiben G, Hunsberger M, et al. Bidirectional associations between psychosocial well-being and adherence to healthy dietary guidelines in European children: prospective findings from the IDEFICS study. *BMC Public Health.* 2017;17(1):926.
4. Ravens-Sieberer U, Bullinger M. KINDL-R. Questionnaire for measuring health-related quality of life in children and adolescents. https://www.google.com/url?sa=t&rct=j&q=&esrc=s&source=web&cd=&ved=2ahUKEwjU_dfU9dPzAhXmwosKHUihADoQFnoECAkQAQ&url=https%3A%2F%2Fwww.kindl.org%2Fapp%2Fdownload%2F6175836884%2FManEnglish.pdf%3Ft%3D1338645880&usg=AOvVaw3gi7cEDCIXVGDYYpQcAagg. Published 2000. Accessed October 18, 2021.
5. Bullinger M, Brütt AL, Erhart M, Ravens-Sieberer U. Psychometric properties of the KINDL-R questionnaire: results of the BELLA study. *Eur Child Adolesc Psychiatry.* 2008;17 Suppl 1:125-132.

Table S1. Cross-sectional association between family structure and the children's BMI z-scores in the full sample, adjusted for income and in IDEFICS children only

	Full sample basic covariates set[a] N=7,804		Adjusted for parental income[b] N=6,452		IDEFICS children only[a] N=5,840	
	**β (95% CI)**	**p-value**	**β (95% CI)**	**p-value**	**β (95% CI)**	**p-value**
**Parental structure**						
Two-parent bio. family	Ref.		Ref.		Ref.	
Single parent family	0.09 (0.001, 0.18)	0.048	0.07 (-0.03, 0.17)	0.19	0.10 (0.01, 0.20)	0.029
Blended family	0.07 (-0.04, 0.17)	0.20	0.03 (-0.08, 0.14)	0.64	0.08 (-0.02, 0.19)	0.122
**Number of siblings**	-0.07 (-0.10, -0.03)	<0.001	-0.05 (-0.09, -0.02)	0.006	-0.07 (-0.11, -0.03)	<0.001

Abbreviations: β effect estimate, CI confidence interval, bio. biological, Ref. reference category

[a]Mixed-effects linear regression model with parental structure, number of siblings, sex, age, parental education as independent variables, while country and family are random effects. The results are shown only for parental structure and number of siblings.

[b]Mixed-effects linear regression model with parental structure, number of siblings, sex, age, parental education and parental income as independent variables, while country and family are random effects. The results are shown only for parental structure and number of siblings.

Table S2. Cross-sectional association between number of siblings and the children's metabolic score, stratified by parental structure

	Two-parent bio. family n = 2,873		Single-parent family n = 353		Blended family n = 296	
	β (95% CI)	p-value	β (95% CI)	p-value	β (95% CI)	p-value
**Number of siblings[a]**	-0.01 (-0.15, 0.13)	0.89	-0.73 (-1.14, -0.32)	<0.001	-0.46 (-0.85, -0.06)	0.022

Abbreviations: bio. biological, β effect estimate, CI confidence interval

[a]Mixed-effects linear regression model with number of siblings, sex, age, parental education as independent variables and country and family as random effects. The results are shown only for number of siblings.

Table S3. Cross-sectional association between family structure and the children's metabolic score

	**Full sample n = 3,522**		**Only children n = 493**		**Siblings n = 3,029**	
	**β (95% CI)**	**p-value**	**β (95% CI)**	**p-value**	**β (95% CI)**	**p-value**
**Parental structure**[a]						
Two-parent bio. family	Ref.		Ref.		Ref.	
Single-parent family	0.16 (-0.19, 0.51)	0.37	1.04 (0.39, 1.69)	0.002	-0.33 (-0.75, 0.09)	0.12
Blended family	0.32 (-0.06, 0.71)	0.10	0.83 (-0.04, 1.69)	0.06	0.17 (-0.26, 0.60)	0.44
**Number of siblings**[a]	-0.14 (-0.26, -0.01)	0.031	-	-	-	-

Abbreviations: β effect estimate, CI confidence interval, bio. biological, Ref. reference category

[a] Mixed-effects linear regression model with parental structure, number of siblings (excluded in only children-stratified subgroups), sex, age, parental education as independent variables, while country and family are random effects. The results are shown only for parental structure and number of siblings.

Table S4. Cross-sectional association between family structure and the children's metabolic score, adjusted for BMI z-scores

	**Full sample basic covariates set[a]** n = 3,522		**Full sample adjusted for BMI z-scores[b]** n = 3,522	
	**β (95% CI)**	**p-value**	**β (95% CI)**	**p-value**
**Parental structure**				
Two-parent bio. family	Ref.		Ref.	
Single-parent family	0.16 (-0.19, 0.51)	0.37	-0.12 (-0.36, 0.12)	0.34
Blended family	0.32 (-0.06, 0.71)	0.10	0.11 (-0.16, 0.37)	0.43
**Number of siblings**	-0.14 (-0.26, -0.01)	0.031	0.01 (-0.08, 0.10)	0.82

Abbreviations: β effect estimate, CI confidence interval, bio. biological, Ref. reference category

[a]Mixed-effects linear regression model with parental structure, number of siblings, sex, age, parental education as independent variables, while country and family are random effects. The results are shown only for parental structure and number of siblings.

[b]Mixed-effects linear regression model with parental structure, number of siblings, sex, age, parental education and BMI z-scores as independent variables, while country and family are random effects. The results are shown only for parental structure and number of siblings.

Table S5. Distribution of the additional covariates in the longitudinal sample

**Baseline characteristics**	**Sibling status at baseline**		**Parental structure at baseline**		**Full sample**
	**Only children**	**Siblings**	**Single-parent family**	**Two-parent family**	
**n (%)**	983 (17.49)	4,638 (82.51)	995 (17.70)	4,626 (82.30)	5,621 (100)
**Well-being, mean ±SD (95% CI)**	n = 905<br>39.75 ±4.67<br>(39.45, 40.06)	n=4,245<br>39.68 ±4.66<br>(39.54, 39.82)	n = 887<br>38.44 ±5.02<br>(38.11, 38.77)	n=4,263<br>39.95 ±4.54<br>(39.82, 40.09)	n=5,150<br>39.69 ±4.66<br>(39.57, 39.82)
**Screen time, mean ±SD (95% CI)**	n = 938<br>11.41 ±7.46<br>(10.93, 11.88)	n=4,377<br>11.85 ±7.39<br>(11.63, 12.07)	n = 905<br>12.98 ±8.14<br>(12.45, 13.51)	n=4,410<br>11.52 ±7.21<br>(11.31, 11.73)	n=5,315<br>11.77 ±7.40<br>(11.57, 11.97)
**Healthy diet score, mean ±SD (95% CI)**	n = 930<br>20.79 ±8.33<br>(20.26, 21.33)	n=4,250<br>21.58 ±8.90<br>(21.31, 21.85)	n = 883<br>19.97 ±8.27<br>(19.42, 20.52)	n=4,297<br>21.74 ±8.88<br>(21.48, 22.01)	n=5,180<br>21.44 ±8.81<br>(21.20, 21.68)
**Sport club member, n(%)**					
Yes	416 (42.32)	2,117 (45.64)	386 (38.79)	2,147 (46.41)	2,533 (45.06)
No	529 (53.81)	2,359 (50.86)	551 (55.38)	2,337 (50.52)	2,888 (51.38)
Missing	38 (3.87)	162 (3.49)	58 (5.83)	142 (3.07)	200 (3.56)

Abbreviations: n number of observations, SD standard deviation, CI confidence interval

Table S6. Longitudinal association of a change in parental structure and in sibling status with children's overweight/obesity at follow-up

	**Full sample n = 5,621**	
	**OR (95% CI)**	**p-value**
**Change in parental structure[a]**		
Stable two-parent	Ref.	
Stable single-parent	1.82 (1.24, 2.68)	0.002
Change from two to single parent family	1.21 (0.83, 1.76)	0.32
Change from single to two-parent family	1.14 (0.88, 1.47)	0.32
**Change in sibling status[a]**		
Stable sibling	Ref.	
Stable only child	0.97 (0.74, 1.28)	0.84
Getting a sibling	1.16 (0.82, 1.64)	0.41
Becoming an only child	1.41 (0.92, 2.16)	0.12

Abbreviations: OR odds ratio, CI confidence interval, Ref. reference category

[a]Mixed-effects logistic regression model with change in parental structure, change in sibling status, sex, age at baseline, parental education, BMI z-score at baseline and follow-up time as independent variables, while country and family are random effects. The results are shown only for parental structure and number of siblings.
